# Diamond synthesis from carbon nanofibers at low temperature and low pressure

**DOI:** 10.1038/srep13879

**Published:** 2015-09-09

**Authors:** Chengzhi Luo, Xiang Qi, Chunxu Pan, Wenge Yang

**Affiliations:** 1 School of Physics and Technology, and MOE Key Laboratory of Artificial Micro- and Nano-structures, Wuhan University, Wuhan 430072, China; 2 Laboratory for Quantum Engineering and Micro-Nano Energy Technology, Faculty of Materials and Optoelectronic Physics, Xiangtan University, Xiangtan 411105, China; 3 Center for Electron microscopy, Wuhan University, Wuhan 430072, China; 4 Center for High Pressure Science and Technology Advanced Research (HPSTAR), 1690 Cailun Road, Bldg 6, Shanghai 201203, China; 5 High Pressure Synergetic Consortium (HPSynC), Carnegie Institution of Washington, 9700 S. Cass Avenue, Argonne, IL 60439, USA

## Abstract

In this article, we report a new route to synthesize diamond by converting “solid” carbon nanofibers with a Spark Plasma Sintering system under low temperature and pressure (even at atmospheric pressure). Well-crystallized diamond crystals are obtained at the tips of the carbon nanofibers after sintering at 1500 °C and atmospheric pressure. Combining with scanning electron microscopy, transmission electron microscopy, electron-energy loss spectroscopy and Raman spectroscopy observations, we propose the conversion mechanism as follows: the disorder “solid” carbon nanofibers → well crystallined carbon nanofibers → bent graphitic sheets → onion-liked rings → diamond single crystal → the bigger congregated diamond crystal. It is believed that the plasma generated by low-voltage, vacuum spark, via a pulsed DC in Spark Plasma Sintering process, plays a critical role in the low temperature and low pressure diamond formation. This Spark Plasma Sintering process may provide a new route for diamond synthesis in an economical way to a large scale.

Diamond has broad applications with its wide range of extreme properties^1^. Many approaches have been pursued to make diamonds, such as chemical-vapor-deposition (CVD)^2^, shock-wave processes^3^, plasma activation^4^, high pressure^5^, exotic precursors^6^,^7^ and explosive mixtures^8^, etc., since the first report of diamonds synthesized through high-pressure and high-temperature (HPHT) process^9^. The conversion of graphite into diamond is of considerable technical interest and therefore remains an exciting field in both experimental and theoretical studies. Although graphite can be converted to diamond, the high temperature and high pressure are needed or the catalysts are presence.

Carbon nanotubes (CNTs) could also transform to diamond under different conditions, e.g. laser irradiation-induced^10^, hydrogen plasma post treated^11^, chemical vapor deposition (CVD) by nanotube coating^12^, shock wave^13^, and direct transformation under HPHT^14^. However, the above approaches have serious limitation from low production volumes, low yield and high costs. Here we report a new route to synthesize diamond, which is converted from “solid” carbon nanofibers (CNFs) in a Spark Plasma Sintering (SPS) system under low temperature and low pressure (even at atmospheric pressure) conditions.

The as-grown “solid” CNFs were synthesized from ethanol flame following the process reported before^15^,^16^,^17^. The “solid” CNFs were then treated in SPS system under atmospheric pressure at 1500 °C in a vacuum (mechanical pump) for 5 minutes, as described in the Methods section.

The starting “solid” CNFs were checked with electron microscopy. As shown in Fig. 1a, the CNFs were entangled with each other with diameter between 20 nm and 100 nm, and length in several micrometers. The detailed transmission electron microscopy (TEM) observations revealed that all CNFs had “solid” microstructures (Fig. 1b,c). Obviously, the CNFs were amorphous and composed of small sized and disordered graphitic sheets with short-range order but long-range disorder. The Electron energy loss spectrum (EELS) profile of as-grown CNFs (Fig. 1d) was similar to that of amorphous carbon^18^, which suggested the amorphous structures in the “solid” CNFs.

After SPS treatment, the morphologies of the CNFs were changed largely. The SEM images, as shown in Fig. 2, demonstrated transformation of the pristine tangled CNFs bundles (Fig. 1a) to melting and clustering CNFs (Fig. 2a), agglomerated particles (Fig. 2b), diamond crystals (Fig. 2c), and nanoscale size plates (Fig. 2d). The nanoscale plates, as shown in Fig. 2d, particularly resembled those previously observed for the O- and T-phased phases of the HTHP-polymerized C_60_^19^ and the morphology of the HTHP-polymerized single-wall carbon nanotubes (SWCNTs)^20^. The agglomerated particles and melting CNFs shown in Fig. 2a,b were presumably sintered and coalesced from single crystals during the SPS process. Actually, Zhang and Shen *et al.* obtained the same structures when used CNTs as starting material^21^,^22^. They showed that the SPS process caused the bonding in CNTs to transform from vander Waals in a rope or yarn, to robust sp2, then to sp3 in micro-diamonds. It was significant to note that there were not only diamond single crystals, but also agglomerated particles in their sintered compacts. The largest single diamond crystal was seen to be about 70 μm; and the largest congregated diamond crystal was about 100 μm. The agglomerated diamonds shown in their experiment were presumably sintered, coalesced, and bonded from single crystals during the SPS process, as the SPS promoted the agglomeration of single crystal diamonds. Similar to their work, the present SPS treatment would also promote the agglomeration of crystal diamond in our experiment.

We have conducted high-resolution transmission electron microscopy (HRTEM) observations on a single CNF, as shown in Fig. 3. As shown in Fig. 3(a), the SPS-treated CNFs could be divided into four sections according to the differences of morphologies: A) the section of well graphited, B) the section of strip-shaped, C) the section of onion-liked, and D) the section of diamond-liked.

The A section was defined as that the CNFs could not have remarkable changes, but the graphitic layers became continuous and orderly arranged, the degree of graphitization were improved (Fig. 3b). The B section (Fig. 3c) was quite different from the A section and as-grown CNFs. The B section was the strip-shaped crystalline segment that composed of continuous and stiff graphitic layer structure. The crystalline segment was not parallel to the central axis of CNF but had an angle along the axis, as shown in Fig. 3a. The EELS profile of the B sections (Fig. 3c inset) showed that the π-electron plasmon peak at 285 eV was intensified and the core-loss spectrum was very similar to that of graphite, which implied that the B section has well-crystallized graphite structure.

The structure of the C section (Fig. 3d) was onion-liked, wherein the graphitic layers bended greatly and formed a ring-shaped structure. The D section (Fig. 3e,f) was a well-crystallized particle, which was enveloped by the graphitic shells. The electron diffraction pattern of the crystalline particle (Fig. 3e inset) can be indexed as the face-centered cubic (FCC) structure, with lattice parameter 0.3575 nm, which was very close to the diamond (FCC structure with lattice parameter 0.3566 nm). Meanwhile, the HRTEM image showed that the lattice spacing between neighboring planes of the crystal was about 0.18 nm (Fig. 3f), very close to the (002) plane lattice spacing of FCC diamond (0.178 nm).

Figure 4a showed a comparison of X-ray diffraction (XRD) patterns of the as-grown CNFs and the SPS-treated CNFs. Remarkable differences arose from these data included that: 1) the peaks of iron oxide (Fe_2_O_3_ and Fe_3_O_4_) disappeared after SPS treatment, instead, the body-centered cubic (BCC) phase of pure iron appeared; 2) three strong diamond peaks [(111), (220) and (311)] were observed after SPS treatment clearly; 3) the noisy background was diminished after treatment. The first feature possibly resulted from the reduction effect of carbon on iron oxide. And the latter features showed that the strong effect of SPS treatment on the as-grown “solid” CNFs which resulted in more graphitized structures and diamond formation.

Further Raman spectra measurements confirmed the XRD results and revealed the graphitizing of CNFs and the formation of diamond structure. By comparing the Raman spectra (Fig. 4b) of the as-grown CNFs and that of SPS-treated CNFs, it was found that the characteristic Raman shift of diamond phase appeared at 1332 cm^−1^ while as-grown CNFs had the D band peak at 1325 cm^−1 ^22. This result further confirmed that the “solid” CNFs were converted to the diamond. The G band peak of Raman shift, corresponding to an E_2g_ mode of graphite and relating to vibration of the *sp*^2^ banded carbon atoms in a two-dimensional graphitic hexagonal lattice, also increased from 1572 cm^−1^ to 1583 cm^−1^, implied that the graphite phase also formed from the SPS-sintered CNFs.

For the above experimental results, we can confirm that the diamond grains were successfully synthesized from the “solid” CNFs in SPS. The SEM, TEM and HRTEM observations directly revealed the microstructure changes and the converting processes of the CNFs to diamond during the SPS treatment. Meanwhile, besides the four sections with different morphologies as described above, there were other interesting finding, such as: 1) the microstructure transformation was usually happened at the tip of CNFs; 2) the graphitic layers of CNFs broke by using an axial pressure in SPS, and the length of CNFs decreased with the increase of pressure; 3) there were sometimes several smaller diamond particles formed in one CNF, while for most cases only one diamond particle was formed in a CNF.

Based on the above microstructure observations, we suggested the conversion processes from the “solid” CNFs to diamond as follows: the disorder “solid” CNFs → well crystallined CNFs → bent graphitic sheets → onion-liked rings → diamond single crystal → the bigger congregated diamond crystal.

We proposed a model for elucidating the conversion of the “solid” CNFs to diamond. SPS technique is a pressure-assisted method based on a high-temperature plasma (spark plasma) momentarily generated in the gap between powder material by electrical discharge during on-off d.c. pulse. It has been suggested that the d.c. pulse could generate several effects, such as spark plasma, spark impact pressure, Joule heating, and electrical field diffusion effect^23^. When the “solid” CNFs, as one-dimensional materials which had high length-to-diameter ratio, were treated in SPS, spark plasma and high localized temperatures could be momentarily generated by a high-energy, low-voltage sparking pulse-current at the tip of a CNF. Therefore, the tip could be a source of heat, and the thermal energy diffused along the axis of CNF.

Under high temperature, the CNFs would absorb energy; the disordered graphitic sheets with small size in the interior were in metastable state and enhanced the ordering of crystal lattice. Therefore, the as-prepared CNFs, which was amorphous and composed of small sized and disordered graphitic sheets, converted to ordered and crystalline segment after SPS treatment. Meantime, at high temperature, and in the presence of plasmas during SPS, the CNFs would absorb energy, leading to the breakage of C–C bonds with high defect density and high energy, and thus the CNF tip would collapse at one point. To reduce the surface energy, the broken carbon domains tended to close from inside to outside, and resulted in the formation of the carbon nano-onions. Due to smaller radius and lower energy level contained at the outer layers, these broken carbon domains could not be converted into an associated crystal form, and left the carbon onions sheathed by an amorphous layer. The formation of high curvature of the graphitic shells in the onion-like ring leaded to an increased fraction of *SP*^3^ bonds, which facilitated the transformation of diamond. Zheng *et al.*^24^ also found the similar microstructure changes of CNFs, when the CNFs were heat-treated at temperature of 2500 °C.

Moreover, with the presence of mass plasmas in the gap between the tips of neighborhood CNFs, the onion-liked rings could be converted to diamond with the assistant of the energy from SPS. The role of plasmas to the diamond conversion from CNFs might be similar to that using electron beam by Banhart *et al.*^25^. In addition, the high localized temperature at the tip and the remarkable stress gradient, which were created by the thermal gradient occurring in the tip zone^26^, might play a key role to the conversion. As a result, the onion-liked ring could be transform to the diamond single crystal.

SPS process has been commonly used to consolidate refractory metals and functionally graded materials which are hard to densify by conventional sintering methods. For the melting, clustering and agglomerating of the CNFs in SPS system, the diamond single crystal could be coalesced and bonded to form bigger diamond crystal. However, we have to point out that we do not know whether the iron catalyst particles played a catalysis role on the conversion from the CNFs to diamond during SPS treatment. Unfortunately, we failed to synthesize diamond by using pure CVD carbon nanotubes with the same treated techniques. These indicated that the “solid” CNFs with amorphous microstructure were of a stronger intension to convert to diamond than the “hollow” CNTs with well-crystallized microstructure. Zhang *et al.*^21^ successfully synthesized diamond from CVD-prepared CNTs in SPS, and however, their experiment did not provide the transformation procedure. In the present work, the conversion process of the CNFs to diamond was systematically revealed by TEM and HRTEM, and we believe that our finding could provide a clear guideline for synthesizing diamond from CNTs and CNFs.

In conclusion, we described the conversion of “solid” CNFs to diamond at low temperature and pressure in a SPS system. We also put forward a new model for the conversion. As a newly developed low-temperature and pressure assisted rapid sintering technique, SPS was originally designed for simple, cost effective, rapid, routine synthesis of new materials, it may provide a new route for diamond synthesis on a large scale.

## Methods

We have previously shown that the process of synthesizing solid carbon nanofibers (CNFs) from ethanol flames^15^,^16^,^17^. The experiment utilized the common combustion apparatus for ethanol flames. The fuel was absolute ethanol (C_2_H_5_OH). The size of flame was about 25 mm in diameter in the middle and 80 mm in height with 15 mm wick. Carbon steels and low alloy steels, such as Q235 (commercial pure Fe), 45# (0.45%C steel), T8 (0.80%C steel), 40Cr, 16Mn, 65Mn, were used as the substrates. First, the sampling surface of the substrates was mechanically polished to certain roughness by using different grade of abrasive papers. Then, the surfaces were pretreated by dipping in a solution of 5 ml pure nitric acid (HNO_3_) + 100 ml absolute ethanol (C_2_H_5_OH) for several seconds. The purpose of this process was supposed to generate tiny particles on the surface for catalyzing growth of CNFs. The total synthesizing conditions was not strictly limited. It means that the present CNFs may very easily be synthesized on substrate in an ethanol flame. At last, the modified surface was faced down against the flame when it was inserted into the central core. The temperature at the surface was maintained around 760 °C.

The as-grown CNFs were treated according to the following process: 1) CNFs were heat-treated at temperature 600 °C for 2 hours. 2) Then the CNFs were treated in a spark plasma sintering system (SPS-3.20MK-II, Sumitomo Heavy Industries, Japan) under atmospheric pressure at 1500 °C in a vacuum (mechanical pump) for 5 min. The temperature was monitored with an optical pyrometer focused on a hole (1.5 mm in depth and 0.3 mm in diameter) in the graphite die. The heating rate was maintained at 100 K min^−1^ and the applied direct current was about 1000 A (voltage <5 V), with a pulse duration of 12 ms and a pulse interval of 2 ms.

The morphology and microstructure of the samples were characterized using a scanning electron microscope (SEM) (FEG-SEM, SIRION, FEI, Netherlands) and high resolution transmission electron microscope (HRTEM) (FEG-HRTEM, JEM-2010FEF, JEOL, Japan) with an electron-energy loss spectroscopy (EELS) accessory. Raman measurement was conducted in a Raman spectroscopy (LabRAM HR 800 UV, HPRIBA JOBIN YVON, France) with a He-Ne laser excited at 632.8 nm. The structure of specimens were analyzed using X-ray Diffractometer (XRD, D8 ADVANCE, Bruker AXS, Germany) using Cu Ka radiation (λ = 0.15406 nm) at a scanning rate of 0.02° s^−1^.

## Additional Information

**How to cite this article**: Luo, C. *et al.* Diamond synthesis from carbon nanofibers at low temperature and low pressure. *Sci. Rep.* **5**, 13879; doi: 10.1038/srep13879 (2015).

## Figures and Tables

**Figure 1:**
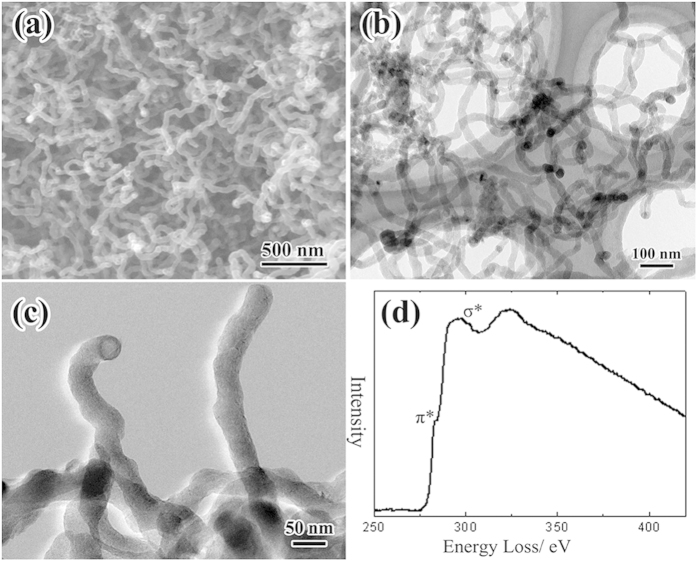
Microstructure characterizations of the original CNFs. (**a**) SEM morphology of the CNFs. (**b**) TEM and (**c**) HRTEM morphologies of the “solid” CNFs. (**d**) EELS profile of the as-grown CNFs.

**Figure 2:**
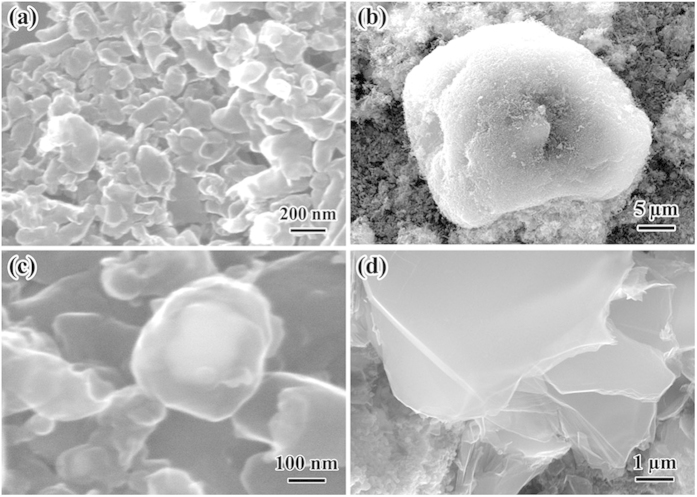
SEM morphologies of CNFs after SPS treatment. (**a**) melting and clustering CNFs. (**b**) Agglomerated particles. (**c**) Diamond crystals. (**d**) Nanoscale size plates.

**Figure 3:**
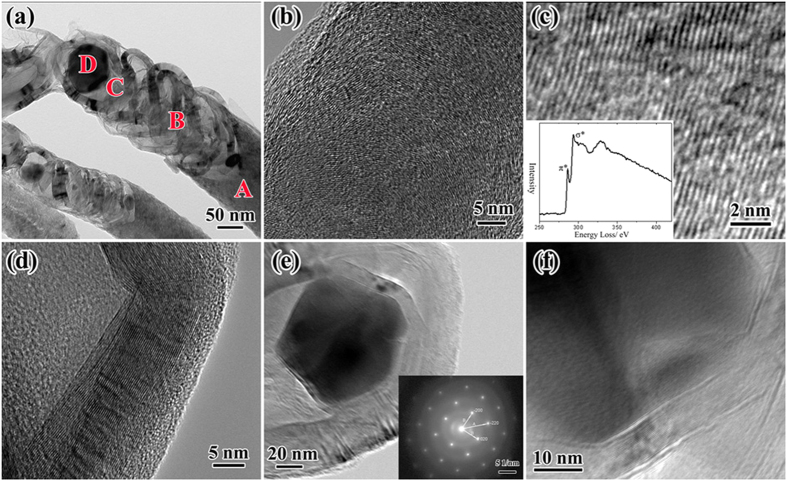
Microstructure Characterization of CNFs after SPS treatment at 1500 °C and atmospheric pressure for 5 min. (**a**) Typical TEM image of the SPS-treated CNFs. We can divide the SPS-treated CNFs into four sections according to the difference of morphologies, as labeled with A, B, C and D. (**b**) HRTEM image of A section. (**c**) HRTEM image of B section, the inset shows the EELS profile of B section. (**d**) HRTEM image of C section. (**e**) HRTEM image of D section, the inset shows the selected-area electron diffraction pattern from the particle in D section. (**d**) HRTEM image of the region in Fig. 3(e).

**Figure 4:**
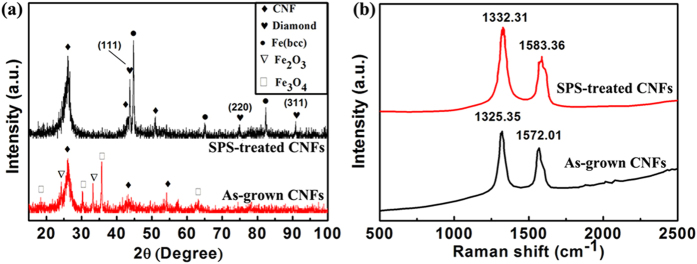
X-ray diffraction pattern and Raman Spectra of the as-grown CNFs and SPS-treated CNFs. (**a**) X-ray diffraction pattern. (**b**) Raman Spectra. SPS-treatment was conducted at 1500 °C and atmospheric pressure for 5 min.
